# Quantifying the sensory and emotional perception of touch: differences between glabrous and hairy skin

**DOI:** 10.3389/fnbeh.2014.00034

**Published:** 2014-02-11

**Authors:** Rochelle Ackerley, Karin Saar, Francis McGlone, Helena Backlund Wasling

**Affiliations:** ^1^Department of Physiology, University of GothenburgGothenburg, Sweden; ^2^Clinical Neurophysiology, Sahlgrenska University HospitalGothenburg, Sweden; ^3^School of Natural Sciences and Psychology, Liverpool John Moores UniversityLiverpool, UK

**Keywords:** affective touch, c-tactile, discriminative touch, pleasant, psychophysics, somatosensory

## Abstract

The perception of touch is complex and there has been a lack of ways to describe the full tactile experience quantitatively. Guest et al. (2011) developed a Touch Perception Task (TPT) in order to capture such experiences, and here we used the TPT to examine differences in sensory and emotional aspects of touch at different skin sites. We compared touch on three skin sites: the hairy arm and cheek, and the glabrous palm. The hairy skin contains C-tactile (CT) afferents, which play a role in affective touch, whereas glabrous skin does not contain CT afferents and is involved in more discriminative touch. In healthy volunteers, three different materials (soft brush, sandpaper, fur) were stroked across these skin sites during self-touch or experimenter-applied touch. After each stimulus, participants rated the tactile experience using descriptors in the TPT. Sensory and emotional descriptors were analyzed using factor analyses. Five sensory factors were found: Texture, Pile, Moisture, Heat/Sharp and Cold/Slip, and three emotional factors: Positive Affect, Arousal, and Negative Affect. Significant differences were found in the use of descriptors in touch to hairy vs. glabrous skin: this was most evident in touch on forearm skin, which produced higher emotional content. The touch from another was also judged as more emotionally positive then self-touch, and participants readily discriminated between the materials on all factors. The TPT successfully probed sensory and emotional percepts of the touch experience, which aided in identifying skin where emotional touch was more pertinent. It also highlights the potentially important role for CTs in the affective processing of inter-personal touch, in combination with higher-order influences, such as through cultural belonging and previous experiences.

## Introduction

Tactile experiences give rise to traditionally-viewed discriminative touch sensations felt on our skin; however, sensations also arise that relate to emotional aspects of these tactile events. Few attempts have aimed at establishing a comprehensive language describing this full tactile experience, which is inevitably a combination of sensory and emotional processing. Typically, researchers explore only one or two dimensions of touch, for example, using a visual analog scale with two endpoints for attributes such as roughness or pleasantness (Essick et al., 1999; Guest et al., 2009; Löken et al., 2009; Libouton et al., 2010). A similar lack of descriptive language existed for describing painful sensations subjectively, until the McGill pain questionnaire was devised to capture multi-dimensional (sensory/discriminative to affective/motivational) aspects of pain (Melzack and Torgerson, 1971). There has been a parallel need to subjectively report the multi-dimensional nature of tactile experiences, using more encompassing terminology and methods. Previous attempts at quantifying the perceptual dimensions that form the basis of tactile experiences have included multi-dimensional scaling of texture perception, which has revealed the main textural dimensions as including “Rough-Smoothness” (Hollins et al., 1993, 2000; Picard et al., 2003; Guest et al., 2011), “Hard-Softness” (Srinivasan and LaMotte, 1995; LaMotte, 2000), “Sticky-Slipperiness” (Hollins et al., 2000), and additionally, “Hot-Cold” and “Dry-Wet”(Guest et al., 2011).

The exploration of the emotional experience of touch has been given less attention, but this is of importance considering the significance of touch in social interaction, grooming behaviors and well-being (Morrison et al., 2010). Part of the rewarding (pleasant) properties of touch are hypothesized to be provided through the activation of low-threshold, slowly-conducting, unmyelinated C-tactile (CT) afferents in the skin (Olausson et al., 2010). These afferents are found exclusively in hairy skin surfaces (e.g., face: Nordin, 1990; arm: Vallbo et al., 1993, 1999), and not in the glabrous skin (e.g., palm; Olausson et al., 2010), which contains no hairs and has a much thicker epidermal layer (McGlone and Reilly, 2010). They are understood to directly encode pleasant aspects of tactile experiences in the periphery, although the emotional relevance of touch is also processed centrally in more frontal regions of the brain (Rolls et al., 2003; McGlone et al., 2012). Research into CT afferents has given rise to the “affective touch hypothesis,” which predicts that the role of the CT system is to provide or support emotional and behavioral responses in skin-to-skin contact between individuals (Olausson et al., 2010). CT afferents respond optimally to slowly-moving touch and their firing frequency correlates with psychophysical ratings of pleasantness (Löken et al., 2009). Additionally, CT afferents have been shown to project to the posterior insular cortex (Olausson et al., 2002, 2008; Morrison et al., 2011), a region known for affective processing (Craig, 2011).

There is a need for methods to investigate the full range of sensations produced during a variety of tactile experiences. Guest et al. (2011) described the development and validation of a descriptive scale for touch perception, where participants rated 262 touch-related English adjectives on how well they described aspects of touch. The work resulted in a Touch Perception Task (TPT) consisting of 26 sensory and 14 emotional descriptors that can be used to uncover how different aspects of touch are associated with specific tactile events. Guest et al. (2011) demonstrated the usefulness of the TPT by testing it in an experimental setting. They found that discriminative (sensory) and emotional (affective) touch can differ in quality according to the skin site touched, as well as who and what is doing the touching. McGlone et al. (2012) used a simplified version of the TPT to discern differences in sensory and emotional aspects of touch to hairy and glabrous skin, where subjective ratings along a single dimension (pleasantness) showed no distinctions, demonstrating the usefulness of such an approach. In the present work, we use the TPT and hypothesize that touch on hairy skin will generate more emotional experiences due to increased affective signaling, thus we compare the arm and cheek (CT-innervated skin) with the glabrous skin of the palm (no CTs). We include a self-touch and other-touch condition, and hypothesize that the touch from another will be more emotionally rewarding on hairy skin, as compared to self-touch and touch to glabrous skin. This is based on the prediction that touch to skin containing CT afferents should produce more emotional responses, and that self-touch gives efferent copy feedback, which can have a cancellation effect on the perception of touch. Different materials were included in the design of the experiment to assess how typically-experienced textures are felt over the different skin sites, to test the range of the TPT. We aimed to extend the findings of Guest et al. (2011) by exploring an additional skin site (the cheek, as well as the arm, and palm), and touching the participant with different materials. Guest et al. (2011) used materials that ranged from smooth silky polyester and rough hessian, whereas we presently ranged from smoother fur to rougher sandpaper. Additionally, we aimed to test the reproducibility of the full TPT in a different country and native language (Sweden), to investigate whether we are testing the underlying description of touch, rather than simply language effects.

## Methods

A total of 20 healthy participants completed the experiment (average age 26 years ±6 *SD*; 9 males). They were given standard information about the experiment and written, informed consent was obtained. The investigation conformed to local ethical approval from the University of Gothenburg and was performed in accordance with the Declaration of Helsinki. Participants were paid for participation and the experiment took no more than 1 h. Touch from different materials was investigated at three skin sites, using self-touch from the participant and the touch from another, delivered by the experimenter. The sensations arising from touch were quantified using the TPT (Guest et al., 2011), which comprises 26 sensory and 14 emotional descriptors. The TPT is based on a list of English words that contribute significantly as descriptors for sensory and emotional aspects of touch. It is formulated in English but as the study was conducted in Sweden, the descriptors were translated into Swedish. The translation was carried out independently by three fluent English-speaking, native Swedish individuals, and the descriptors were also compared to dictionary definitions and translations of the words. It was back-translated to English by an individual naïve to the original English version. As the back-translated English words corresponded to the original English version, the translation into Swedish was considered satisfactory. Table 1 details all of the descriptors and the translations. In the task, the degree to which each word described the sensation perceived was rated directly after each touch application. The rating scale consisted of 5 choices: “not descriptive,” “slightly descriptive,” “moderately descriptive,” “highly descriptive,” and “very highly descriptive.” These were also translated into Swedish for use in the TPT.

**Table 1:** **The list of sensory and emotional descriptors constituting the TPT**.

**Sensory descriptors**		**Emotional descriptors**	
**English**	**Swedish**	**English**	**Swedish**
Bumpy	Gropigt	Arousing	Upphetsande
Burning	Brännande	Calming	Lugnande
Cold	Kallt	Comfortable	Bekvämt
Damp	Fuktigt	Desirable	Begärligt
Dry	Torrt	Discomfort	Obekvämt
Firm	Fast	Enjoyable	Trevligt
Fluffy	Fluffigt	Exciting	Spännande
Fuzzy	Luddigt	Irritating	Irriterande
Greasy	Fet	Pleasurable	Njutbart
Gritty	Grynigt	Relaxing	Avslappnande
Hairy	Hårigt	Sensual	Sensuellt
Hard	Hårt	Sexy	Sexigt
Hot	Hett	Soothing	Lindrande
Jagged	Kantig	Thrilling	Nervkittlande
Lumpy	Knöligt		
Prickly	Stickigt		
Rough	Skrovligt		
Rubbery	Gummiaktigt		
Sharp	Vasst		
Slippery	Halt		
Smooth	Lent		
Soft	Mjukt		
Sticky	Klibbigt		
Vibrating	Darrigt		
Warm	Varmt		
Wet	Vått		

*The English TPT was translated into Swedish for use in the present study*.

The tactile experience was manipulated in three ways by using different materials to gently stroke the skin (referred to as the “Material” condition) at different skin sites (referred to as the “Site” condition) and either using self-touch (participants touched themselves with the materials) or other-touch (the experimenter delivered the stimuli; referred to as the “Mode” condition). The experimenter was the same (female) for all participants and was within side-view for both the self-touch and other-touch. She was naïve to the background of the study. The materials used were a rectangular piece of sandpaper (grade: P120; size: 70 × 40 mm; see Verrillo et al. (1999) for classification of the sandpaper), a piece of artificial fur (soft 10 mm long hairs; size 80 × 50 mm) and an artist's brush (size: 60 mm wide; hairs: 30 mm long goat's hair). The sandpaper was chosen as an extreme boundary condition, where the touch would be felt as very different to the soft brush and fur. The brush and fur were used to compare more similar-feeling materials. The fur and the sandpaper were fastened around the experimenter's index and middle fingers while the brush had a short (80 mm) handle which was held by the experimenter during application to the skin. The participant used the materials in the same manner. There was no measurement of force of the application, however the experimenter was trained to deliver soft strokes that did not exceed 0.5 N and made sure that the participants stroked themselves in a similar way. The skin sites used were the left palm, left dorsal forearm and left cheek.

Each stimulus was applied in a randomized order, as determined by a custom-written LabVIEW (National Instruments, Austin, TX) program. This program enabled the experimenter to see the stimulus type to be delivered on a screen in front of them, but this was hidden from the participant. The participant sat comfortably in an examination chair. Stimuli were delivered in proximal to distal direction (except over the cheek which was from near the left eye to near the mouth) at approximately 3 cm s^−1^. Each combination of Mode, Material and Site was delivered once. The participants were allowed to watch the stimuli being applied to the arm and palm as these skin sites were fully visible. The cheek was obviously less visible. After each stimulus delivery, participants rated their perception of the sensation evoked using the TPT, which was presented on an interactive touch screen (iPad; Apple, Cupertino, CA) placed in front of them (cf. Guest et al. (2011) where this was done using pencil and paper). It was presented as four pages, for ease of completion, where each page contained 10 of the descriptors in a set order (see Table 1). The descriptors were located on the left side of the screen and the participant was required to touch a location on the right of the screen that corresponded with how well each descriptor corresponded to the tactile experience (as above, “not descriptive” to “very highly descriptive”). The output from the TPT was recorded by the LabVIEW program and coded into ratings of 0–4, where 0 = not descriptive to 4 = very highly descriptive.

Statistical analyses were carried out with SPSS (version 18; IBM, Armonk, NY). The data were analyzed using similar techniques to Guest et al. (2011). The scores from the sensory descriptors and the emotional descriptors were entered into separate factor analyses to reduce the data into groups of sensory and emotional factors, respectively, as our aim was to separate these aspects of touch (as also consistent with texture perception research; Gámbaro et al., 2002; Guest et al., 2011). However, we tested whether a single factor analysis was feasible and gained very similar factors to the separate tests. Kaiser-Meyer-Olkin measures of sample size adequacy were gained for each factor analysis to verify that a sufficient sample size was used. For both analyses, this measure was >0.9, which is well above the 0.5 acceptability limit (Kaiser, 1974). Bartlett's test was used to check the significance of the correlation matrix of factors and was found to be significant (*p* < 0.001) for both factor analyses, indicating that the descriptors cluster into factors. The extraction method used for each factor analysis was principal axis factoring, where a model is derived from which factors are estimated. This has been shown to be preferable over other extraction methods (Russell, 2002). A number of criteria were used to determine the optimal number of factors to extract. The data were explored with an initial factor analysis and the eigenvalue for each factor was inspected using a scree plot, to graphically determine the importance of each factor. Kaiser (1960) recommends retaining factors with eigenvalues of >1, which was used in the present analysis. We set an additional criterion where included factors must also account for >5% of the variance. The factors in each analysis that satisfied these criteria were explored further.

Factor rotation was used to better differentiation between the factors; independent orthogonal (Varimax) rotation and related oblique (Promax) rotation were tested (as recommended by Russell, 2002). Orthogonality (independence) of factors is an unrealistic assumption, as psychological factors are often correlated, and Field (2009) states that oblique rotation should be used if there is any reason for theoretical relationships between factors. As the factors under investigation related to complex, higher-order tactile concepts, we could not exclude correlations between factors. We tested both types of rotation and the results were similar; however, oblique rotation was chosen as significant correlations were found between the factors (i.e., they were related), in both the sensory and emotional factor analyses. Using oblique Promax rotation was also advantageous because the constraint of independence of factors is relaxed, although it includes orthogonal rotation. The Promax procedure conducts an orthogonal Varimax rotation initially, and then allows correlations between factors to improve the factor fit, resulting in more realistic factors (Fabrigar et al., 1999; Russell, 2002; Schmitt, 2011).

The extracted factors were composed of significantly contributing descriptors (loadings of >0.3; Field, 2009). In both the sensory and emotional analyses, all of the descriptors from that set were used i.e., each descriptor contributed significantly to at least one factor. As oblique rotation was used, a pattern matrix and a structure matrix were generated, containing the loadings of significant descriptors onto each factor, relating to the regression coefficients and correlation coefficients, respectively. The pattern matrix (regression) contains information about the unique contribution of a descriptor to a factor. The structure matrix (correlation) includes the relationships between the factors and is similar to orthogonal rotation output. The scores for each factor were determined on the weightings of significant descriptor contributions to that factor, from inspection of both the pattern and structure matrices to take account of all associations. A repeated measures analysis of variance (ANOVA) was used to explore significant differences in the regression data. This consisted of a 3 × 3 × 2 design where the Material condition had three levels (sandpaper, fur, brush), the Site condition had three levels (palm, arm, and cheek) and the Mode condition had two levels (self-touch and other-touch). Descriptive statistics were calculated and a full factorial model was used to explore the factors and factor interactions. Data were checked for sphericity (i.e., equality in variance in the different levels of the factor) using Mauchly's test and if this was violated, a Greenhouse-Geisser correction was used in quoting the statistics. The main effects of each factor were compared, and the different levels of the factors were contrasted using Bonferroni-corrected *post-hoc* tests, controlling for multiple comparisons. Statistical significances were sought at the *p* < 0.05 level (*p* values are given to three decimal places).

## Results

Analyses of the data reveal differences in the perception of sensory and emotional components of the tactile experience with regard to the skin site stroked (Site), the material used (Material), and the mode used to do this (Mode). We explore the sensory and emotional data individually, as separate factor analyses were conducted on each set of descriptors to separate these aspects of touch (as per Guest et al., 2011). We specifically aimed to relate our results to the underlying neurophysiology of the skin site investigated, i.e., whether it contained CT afferents or not. The names of the factors were chosen according to the descriptors that loaded with high values on that factor from the pattern (regression) and structure (correlation) matrices.

### Sensory descriptors

Factor analysis of the sensory descriptors revealed five factors that contributed significantly to the overall variance in the data, named: “Texture,” “Pile,” “Moisture,” “Heat/Sharp,” and “Cold/Slip.” Table 2 shows the details for each factor, including the loadings (regression and correlation coefficients) for significantly contributing descriptors, where the highest loading descriptors are at the top of the table. The Texture factor accounted for the vast majority of variance in the data (36.7%), where descriptors like “bumpy” and “firm” contributed highly to this factor (loading > 0.8). The other four factors contributed less to the variance of the data but provided additional, significant input to the sensory experience. The effect of gender of the participants was investigated to see whether the factor analysis was changed due to this dimension but no significant differences were found between the groups.

**Table 2:** **Sensory descriptors factor analysis**.

**Factor name**	**Factor 1: Texture**				**Factor 2: Pile**				**Factor 3: Moisture**				**Factor 4: Heat/Sharp**				**Factor 5: Cold/Slip**			
**Variance**	**36.7%**				**11.4%**				**8.2%**				**5.9%**				**5.1%**			
**Output**	**Regression**		**Correlation**		**Regression**		**Correlation**		**Regression**		**Correlation**		**Regression**		**Correlation**		**Regression**		**Correlation**	
Descriptor and loading	Bumpy	0.96	Bumpy	0.89	Fuzzy	1.01	Soft	0.89	Damp	0.77	Greasy	0.70	Hot	0.80	Burning	0.83	Cold	0.74	Cold	0.60
	Lumpy	0.91	Hard	0.87	Fluffy	0.88	Fluffy	0.83	Wet	0.73	Damp	0.69	Burning	0.77	Hot	0.76	Slippery	0.42	Smooth	0.58
	Jagged	0.82	Rough	0.87	Hairy	0.87	Fuzzy	0.82	Greasy	0.70	Wet	0.68	Warm	0.44	Sharp	0.60			Rough	−0.56
	Firm	0.80	Firm	0.80	Soft	0.63	Smooth	0.79	Rubbery	0.48	Rubbery	0.46	Sharp	0.34	Rough	0.50			Soft	0.55
	Dry	0.75	Gritty	0.77	Warm	0.57	Hairy	0.77	Sticky	0.39	Smooth	0.45			Hard	0.49			Slippery	0.53
	Hard	0.71	Jagged	0.77	Smooth	0.45	Rough	−0.75			Soft	0.42			Bumpy	0.46			Gritty	−0.50
	Vibrating	0.65	Soft	−0.75	Vibrating	0.30	Hard	−0.66			Rough	−0.35			Dry	0.43			Hard	−0.43
	Sticky	0.60	Lumpy	0.75			Bumpy	−0.51			Dry	−0.35			Smooth	−0.41			Hairy	0.39
	Gritty	0.60	Smooth	−0.74			Firm	−0.53			Slippery	0.34			Warm	0.41			Firm	−0.38
	Rough	0.56	Sharp	0.72			Gritty	−0.62			Fluffy	0.32			Soft	−0.41			Bumpy	−0.35
	Prickly	0.55	Dry	0.69			Sharp	−0.49							Gritty	0.40			Rubbery	0.33
	Sharp	0.53	Burning	0.57			Jagged	−0.48							Prickly	0.39			Lumpy	−0.31
			Prickly	0.55			Burning	−0.43							Jagged	0.38				
			Fluffy	−0.49			Slippery	0.41							Firm	0.38				
			Vibrating	0.47			Lumpy	−0.38							Vibrating	0.32				
			Hairy	−0.44			Dry	−0.36							Lumpy	0.32				
			Fuzzy	−0.39			Prickly	−0.33												
			Hot	0.34			Warm	0.32												
			Slippery	−0.35			Greasy	0.31												
			Sticky	0.32																

*Five significant factors were found in the sensory descriptors data (those contributing >5% of the variance; detailed in the Methods) and named Texture, Pile, Moisture, Heat/Sharp, and Cold/Slip. The descriptors and their significant loadings (>0.3) are shown for both the regression (pattern matrix) and the correlation (structure matrix) factor analysis output. The regression data shows the unique loadings of descriptors onto each factor i.e., how much a descriptor predicts that factor (regression coefficients). The correlation data shows the loadings of descriptors onto each factor, when taking into account the relationships between the factors i.e., how much a descriptor relates to each factor (correlation coefficients)*.

**Figure 1:**
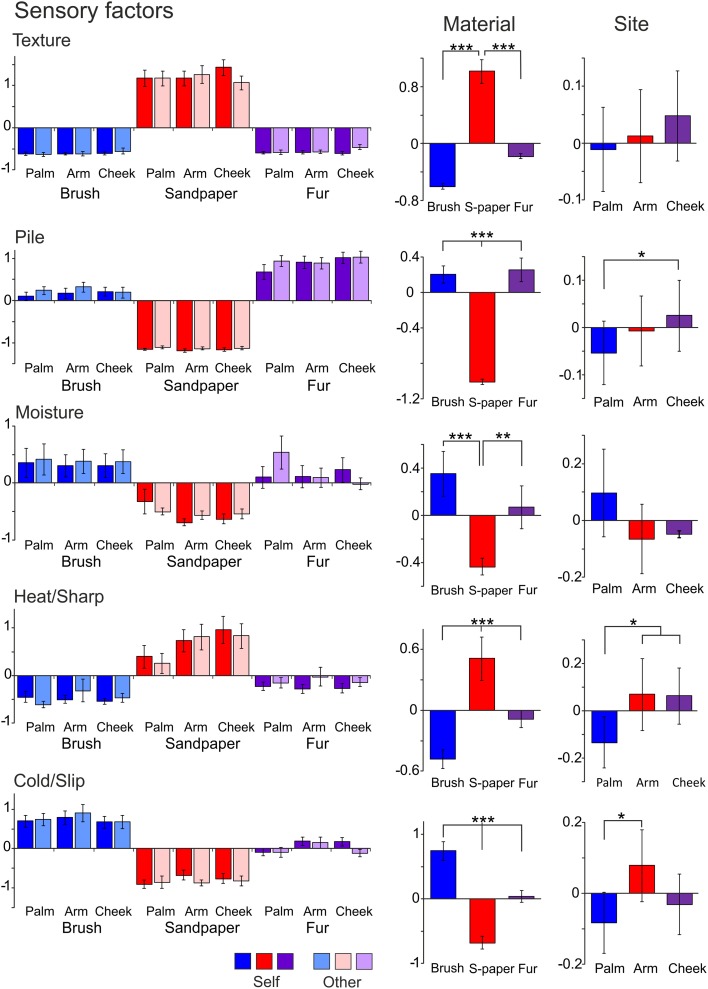
**The average scores for the sensory factors**. Each factor (Texture, Pile, Moisture, Heat/Sharp, and Cold/Slip) is displayed with the average scores for the levels of each condition (left graphs) and the average for the Material and Site conditions on the right side. Significant differences between the levels of conditions showing significant main effects are marked with asterisks and show ^*^p < 0.05, ^**^p ≤ 0.01 or ^***^p ≤ 0.001. For more detail on the significant differences, see Table 3. The materials are shown in blue: brush, red: sandpaper, and purple: fur. Sandpaper is abbreviated to S-paper. Error bars ±1 s.e.m.

In the ANOVA tests, all five factors showed a significant main effect for the Material condition (using sandpaper, brush or fur; Table 3), as would be expected using very different types of material (cf. rough sandpaper with the smooth brush and fur). However, in all but the Texture and Moisture factors, participants also distinguished between the sensory properties of the brush and fur, which were much more similar, showing the sensitivity provided by the TPT. For each factor, we explored how the levels of each condition significantly differed. For the Texture factor, sandpaper was clearly perceived as being very different from the brush and fur. This can be seen in Figure 1 (top graphs) and is reflected in the statistics, where sandpaper was always rated as having significantly more textural properties over the brush and fur. In brief, for the other factors in the Material condition there were significant differences between all of the sandpaper, brush and fur ratings, which can be seen in Figure 1 and Table 3. The noteworthy findings include the fur being experienced as a highly fuzzy and fluffy (Pile), the sandpaper being experienced as dry and hot (low on Moisture and high on Heat/Sharp), presumably due to increased friction, and the brush being experienced as Cold/Slip, due to low friction. For the Site condition (touch at the palm, arm and cheek), three factors showed a significant main effect, namely Pile, Heat/Sharp, and Cold/Slip (Table 3). For the Pile factor, ratings from the cheek were higher than on the palm. For the Heat/Sharp factor, both the hairy skin sites (arm and cheek) gave higher ratings than the palm. For the Cold/Slip factor, the arm gave higher ratings than the palm. The results from the Heat/Sharp and Cold/Slip factors suggest that thermal components of touch may be better sensed on hairy skin, compared to glabrous skin. In the Mode condition, only Pile showed a significant main effect. On inspection of the data, the Pile quality of the material was rated as higher when the experimenter was stroking the participant, as compared to when the participant stroked themselves.

**Table 3:**
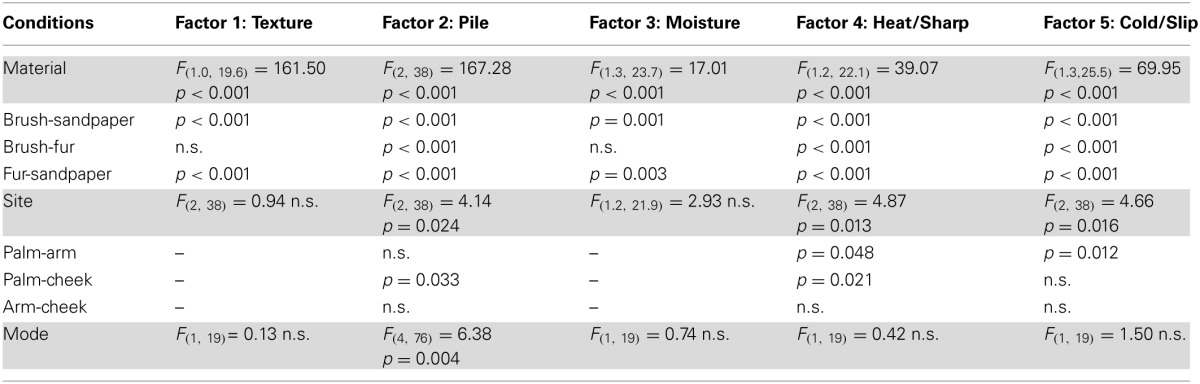
**Significant differences for the condition type in the sensory factors**.

*For each factor (Texture, Pile, Moisture, Heat/Sharp, and Cold/Slip), the main effect for each condition is highlighted in shading and the F value is given with the degrees of freedom; if a significant main effect was found, Bonferroni-corrected post-hoc comparisons were made. Where significant differences were found, the probability (p) values are given unless p < 0.001; n.s. denotes no significant difference*.

### Emotional descriptors

**Figure 2:**
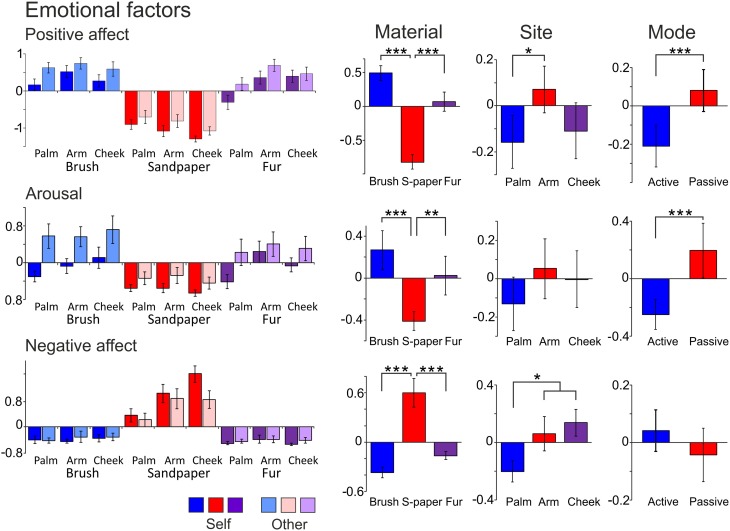
**The average scores for the emotional factors**. Each factor (Positive Affect, Arousal, and Negative Affect) is displayed with the average scores for the levels of each condition (far left graphs), then the average for the Material, Site and Mode conditions. Significant differences between the levels of conditions showing significant main effects are marked with asterisks and show ^*^p < 0.05, ^**^p ≤ 0.01 or ^***^p ≤ 0.001. For more detail on the significant differences, see Table 5. The materials are shown in blue: brush, red: sandpaper, and purple: fur. Sandpaper is abbreviated to S-paper. Error bars ±1 s.e.m.

Factor analysis on the emotional data found three significantly contributing factors to the overall variance, which were named “Positive Affect,” “Arousal,” and “Negative Affect.” Table 4 shows the details of the descriptor loadings for each factor. The Positive Affect factor accounted for the majority of the overall variance in the data (53.2%) and the descriptors “calming,” “relaxing,” “pleasurable,” and “comfortable” all contributed highly to the factor with a loadings of >0.9 (Table 4). The factor names were chosen on the basis of the top-loading descriptors for all of these factors (from the regression and correlation coefficients), as they summed up the descriptors per factor well. Gender sub-groups were explored but no significant differences were found. Both positive and negative emotional factors were found, showing how touch can be experienced in different affective dimensions. Table 5 shows the results of ANOVA statistical tests on the Material, Site and Mode conditions. For the all of the conditions, there was a significant main effect of Material, and for each factor, further significant differences were found between the sandpaper and the brush and between the sandpaper and fur (also see differences in Figure 2). Therefore, the sandpaper was felt as very different i.e., it did not produce positive emotional affect or arousal, rather negative affect, compared to the brush and fur. There was also a significant main effect of Site for all of the factors, which provides insights into the neurophysiology of touch sensations over different skin sites. Stroking on the arm was perceived as significantly more positive in affect than on the palm and cheek. Furthermore, stroking on the arm and cheek were perceived as significantly more negative in affect than on the palm. For the Mode condition, there were significant main effects for the Positive Affect and Arousal factors only. These positive emotional factors showed the same significant difference: that stroking by the experimenter was found to be more evocative than self-stroking.

**Table 4:** **Emotional descriptors factor analysis**.

**Factor name**	**Factor 1: Positive affect**				**Factor 2: Arousal**				**Factor 3: Negative affect**			
**Variance**	**53.2%**				**16.2%**				**7.8%**			
**Output**	**Regression**		**Correlation**		**Regression**		**Correlation**		**Regression**		**Correlation**	
Descriptor and Loading	Relaxing	0.97	Relaxing	0.94	Sexy	0.95	Sexy	0.87	Irritating	0.92	Irritating	0.94
	Calming	0.97	Pleasurable	0.94	Arousing	0.85	Arousing	0.82	Discomfort	0.83	Discomfort	0.90
	Pleasurable	0.92	Comfortable	0.94	Sensual	0.70	Sensual	0.79			Comfortable	−0.57
	Comfortable	0.90	Calming	0.92	Exciting	0.59	Exciting	0.67			Enjoyable	−0.55
	Enjoyable	0.83	Enjoyable	0.92			Desirable	0.57			Relaxing	−0.51
	Soothing	0.79	Soothing	0.78			Pleasurable	0.53			Calming	−0.49
	Desirable	0.69	Desirable	0.77			Enjoyable	0.49			Pleasurable	−0.47
			Sensual	0.57			Soothing	0.45			Desirable	−0.36
			Discomfort	−0.56			Comfortable	0.45			Soothing	−0.36
			Irritating	−0.51			Relaxing	0.44				
			Arousing	0.40			Calming	0.42				
			Exciting	0.41			Thrilling	0.32				
			Sexy	0.35								

*Three significant factors were found in the emotional descriptors data (those contributing >5% of the variance; detailed in the Methods) and named Positive Affect, Arousal, and Negative Affect. The descriptors and their significant loadings (>0.3) are shown for both the regression (pattern matrix) and the correlation (structure matrix) factor analysis output. The regression data shows the unique loadings of descriptors onto each factor i.e., how much a descriptor predicts that factor (regression coefficients). The correlation data shows the loadings of descriptors onto each factor, when taking into account the relationships between the factors i.e., how much a descriptor relates to each factor (correlation coefficients)*.

**Table 5:**
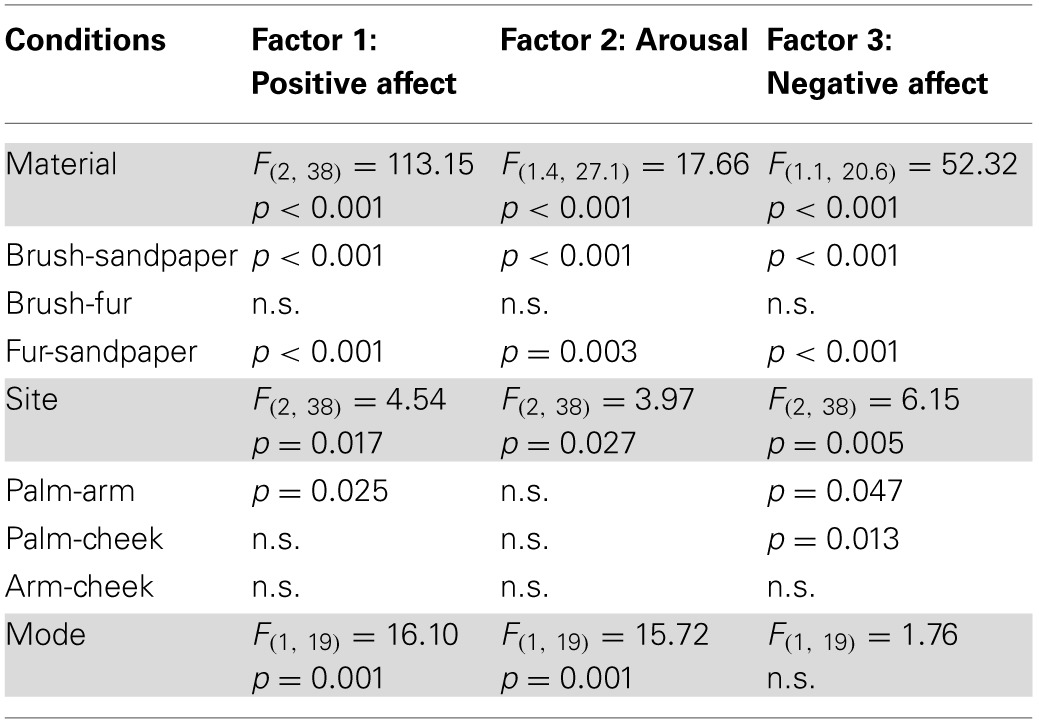
**Significant differences for condition type in the emotional factors**.

*For each factor (Positive Affect, Arousal, and Negative Affect), the main effect for each condition is highlighted in shading and the F value is given with the degrees of freedom; if a significant main effect was found, Bonferroni-corrected post-hoc comparisons were made. Where significant differences were found, the probability (p) values are given unless p < 0.001; n.s. denotes no significant difference*.

## Discussion

Presently, we investigated tactile sensations from stroking three different materials, on three different skin sites, using self- or other-touch. Using the translated TPT, we interpreted the extent to which tactile-related descriptors characterized the touch experience, to give a smaller set of explanatory, higher-level factors. The results show that participants used different sensory and emotional descriptors for self- and other-touch, when touched with different materials, over different skin sites. Our study extends the original TPT work by Guest et al. (2011), particularly the implications for interpreting affective touch. Also, all the descriptors in the current study contributed significantly to at least one factor, therefore we validate the choice of words included in the original TPT as giving a comprehensive description of tactile experiences in general. Similar factors were found in our present TPT and from Guest et al. (2011), showing that there are complex attributes that relate to both the sensory and emotional description of touch. It is likely that the main sensory factor found in both studies, here Texture, and Roughness in Guest et al. (2011), mainly refer to the vibration or frictional qualities of the material touched. We found five contributing sensory factors that described the textural properties of the material, to other attributes such as slip. Previous experiments investigating perceptual tactile space have readily found degrees of roughness, wetness, stickiness, softness and temperature (Hollins et al., 1993, 2000; Picard et al., 2003; Guest et al., 2011). Presently, we found two sensory factors that related to thermal aspects, namely Heat/Sharp and Cold/Slip, but these were intertwined with different tactile attributes. In the original TPT study, Guest et al. (2011) found some thermal qualities; we relate our Heat/Sharp factor to Guest et al.'s Firmness factor, which included descriptors such as “sharp,” “hot,” and “burning,” and our Cold/Slip factor relates well to Guest et al.'s Slip factor, which also included the descriptor “cold.” In the present results, we differentiate between the factors “Moisture” and “Cold/Slip,” which appeared to be separate elements of touch, relating to wetness and thermal smoothness, respectively.

On the other hand, the emotional factor that accounted for the highest variance was Positive Affect here, and Comfort in Guest et al. (2011); these account for more enjoyable aspects of the tactile experience. Arousal was also found as the second emotional factor in both studies. Currently, we found Negative Affect as a third factor, which accounted for the least emotional variance and was driven by the touch of sandpaper; Guest et al. (2011) found no negative affective factor, although they included one rough material. There are little data available on the complexities of affective touch and the findings from both emotional touch descriptor analyses should pave the way for future investigations into the multi-dimensional nature of affective touch. It is of particular interest to investigate affective touch over the body; between the present work and Guest et al. (2011), more is known about affective touch on the upper limbs, which we in part relate to the underlying neurophysiology. Less is known in general about the skin neurophysiology of the torso and lower limbs, and studies could investigate the responses of skin afferents over these areas, as well as their role in affective touch. It is likely that CT afferents, which have been implied in coding pleasant touch (Olausson et al., 2010) are important in signaling affective touch; however, the interpretation of touch is heavily influenced by cognitive, top-down mechanisms (e.g., attention, previous experience, learning) and also the way in which body sites are used (e.g., personal space, social interactions). It is of particular interest to know how the bottom-up input from the skin combines with top-down information to form the percept of affective touch.

The use of modern technology in the present work, for the presentation and collection of the data was advantageous over Guest et al. (2011); using the touch-screen display enabled the participants to fill out the TPT descriptors quickly and focus on the task at hand. As there was no pencil-and-paper coding of the descriptors, this reduced the time taken for analysis and decreased human error. We used a computer program to randomize the order of stimulus presentation (Guest et al.'s was not fully randomized), and this also aided in avoiding human mistakes. There are some differences between the present study and Guest et al.'s (2011), for example, the use of different materials and body sites stroked, and fewer participants in the present work, although both studies demonstrate the scope of interpretation possible in tactile experiences. Comparing the overall results in the present study to Guest et al. (2011), we find that the translated version of the TPT provided related factors; but we could not assume that using the TPT in a different language and cultural would produce similar findings. People belonging to different cultures can have different inter-personal tactile behaviors, for example, people from the United Kingdom, certain parts of northern Europe, and Asia touch each other far less often than those in France, Italy or South America (Jourard, 1966). It was therefore possible that the translated descriptors would not be as descriptive of the tactile experience, as the original (US) English version; however, all descriptors contributed to the tactile sensations. We interpret this as the TPT uncovering the multifaceted sensory and emotional value of touch, which has implications for how tactile information is transmitted to the brain and processed for use in exploratory behaviors and the importance of touch in social situations (Gallace and Spence, 2010).

### Effect of skin site

Both hairy and glabrous skin surfaces contain fast-conducting, low-threshold, myelinated Aβ mechanoreceptive afferents, although at different densities, which readily code discriminative aspects of touch. Hairy skin is additionally innervated by slowly-conducting, unmyelinated CT afferents that are preferentially activated by gentle stroking touch and are hypothesized to signal affective aspects of touch (Löken et al., 2009). We hypothesized that the underlying neurophysiology of skin afferents, particularly CT afferents, would affect the outcome of the verbal description of touch. We find that stroking on arm and cheek (that contain CTs) gives greater affective values than on the glabrous skin of the hand. The differences were especially clear between stroking on the arm and the palm, where the emotional value given to stroking on the palm was less for both the Positive Affect and Negative Affect factors. In essence, this shows that the glabrous palm skin coded much less for the emotional aspects of the tactile stroking. CT afferents are associated with the emotional, rather than discriminative, aspects of touch, although the myelinated mechanoafferent nevertheless deliver tactile information to the brain that can be interpreted in an emotional way. It is certainly true that CTs are not necessary in affective touch, as pleasantness can readily be rated using the fingers (Klöcker et al., 2012). We suggest that CTs aid in the interpretation of affective touch, and their presence in the hairy skin may be related to the role these areas play in receiving touch. The interpretation of all tactile input, combined with other sensory and cognitive factors, will influence how touch is perceived. The only way to investigate the exact role of CTs in emotional touch would be to relate their firing characteristics to psychophysical measures (e.g., Löken et al., 2009), which could be a direction for future studies, especially if in-depth recordings can be made from skin other sites (e.g., thigh, calf).

Although the face is also innervated by CT afferents (Johansson et al., 1988; Nordin, 1990), our findings reveal that touch to the arm was rated as significantly more emotional in a positive manner, than on the cheek. A previous investigation used robot-applied touch and found that pleasantness ratings from hairy skin were significantly higher than from glabrous skin; however ratings from the face were higher than from the arm (Essick et al., 1999). A contributing factor to explain the difference between their findings and the current study is the way in which the stimulus was applied: Essick et al. used a robotic tactile stimulator to deliver stimuli, whereas here stimuli were delivered by the experimenter who was of necessity, close to the participant. Recent work has shown that an object near the eyes enhances a defensive blink reflex due to modulation from higher-order cortical brain areas that code the location of somatosensory stimuli in space (Sambo et al., 2011). This finding is based on visual signals where the person can see or has seen the object in space, and previous studies have shown that visual signals influence tactile perception on body parts that can be seen (Tipper et al., 1998; Haggard et al., 2007; Mirams et al., 2010). Therefore, stimuli entering this peri-personal space, such as touch to the cheek in the current study, may invoke a defensive reaction that is difficult to overcome. Previous work has found that a touch with a potential threatening component affects attention (Poliakoff et al., 2007) and that touch to the face sends particularly strong emotional messages (Lee and Guerrero, 2001). These aspects could underlie the effects we see in our present data, especially as the touch was in view, which differs to Guest et al. (2011)

The sensory descriptors also gave differences between skin sites for some of the factors, namely for Pile, Heat/Sharp and Cold/Slip. The Pile factor encompasses descriptors such as fuzzy, fluffy, and hairy; therefore it appears to account for softer, low force aspects of the stroking stimuli. The ratings for Pile on the cheek were significantly higher than on the palm; it is likely that this shows differences in the sensitivity of these two different skin regions to certain qualities of stimuli, due to differences in both sensory afferent innervation and thickness of skin (McGlone and Reilly, 2010). The skin of the face has been found to be more sensitive to light touch than the glabrous palm skin (Weinstein, 1968; Ackerley et al., 2014), which may relate to the density and type of mechanoreceptive afferents present, as well as the decreased skin thickness. Overall, the lack of CT afferents in the palm does not exclude the ability to perceive pleasantness as studies have shown a range of affective sensations can be perceived using touch input from glabrous skin (Löken et al., 2011; Klöcker et al., 2012; Ackerley et al., 2014). It is of interest to investigate affective touch over more skin sites. Ackerley et al. (2014) find that touch pleasantness does not differ substantially over the body, which is likely due to cognitive factors such as previous experience and memory affecting the interpretation, rather than the density of CT afferents. Therefore, CT afferents may aid in coding affective touch in the periphery, but there are many other factors (e.g., how the body site is used) that influence how touch is actually felt. Future studies could investigate how affective touch is felt over the body, and link this to the usage of the site, as well as skin innervation; it would be possible to conduct the TPT just using the emotional descriptors.

### Effect of application mode

Touch was either applied by the experimenter (other-touch) or by the participant themselves (self-touch). The mode of touch application had an effect on the Positive Affect and Arousal emotional factors, where other-touch was found to give higher ratings than self-touch. The same effect was found for Pile in the sensory factors. Previous work has been contradictory as to whether self-produced touch is experienced as more or less intense that touch from another. Activity in the somatosensory cortex has been found to decrease during self-touch due to movement-related gating (through efference copy) of the somatosensory input (Blakemore et al., 1998, 2000); however, other studies have found increases in somatosensory cortical activity during self-touch (Simões-Franklin et al., 2011; Ackerley et al., 2012), and hypothesize that the motor efference copy cancellation signal in active touch can be countermanded by top-down influences e.g., paying attention. Essentially, the modulation of the cortical processing of touch can depend upon higher-level cognitive processes, such as when a person is exploring an object (typically using the glabrous skin of the hands); it is more useful to countermand the motor cancellation signal so that the features of the object can be better assessed. We found that the emotional factors were particularly susceptible to the mode of touch, as did Guest et al. (2011). This has implications for the understanding of different aspects of touch and how they are interpreted in the brain. Affective touch appears to be especially sensitive when touch is being received, rather than given; this demonstrates the significance of touch in social interactions. As the positive emotional aspects from other-touch were increased over self-touch, the input from CT afferents may not be subject to the same top-down processes that can modulate the processing of the sensory information. Therefore the positive emotional aspects of self-touch are likely always felt as less than other-touch. However, there may be experimenter-interaction confounds. The other-touch stimulation was always applied by the same female experimenter. It is possible that the experimenter added involuntary and uncontrolled social signals, and the Mode-related ratings from the TPT might have been both under- and over-estimated. It has also recently been shown that cortical processing of tactile and visual input can be influenced by the sex of the touching person (Gazzola et al., 2012), and this could therefore affect the perception of touch.

### Effect of material

Three different materials were used to apply the touch: coarse sandpaper, a soft brush and soft artificial fur, and a clear effect of Material was seen for all the factors. In general, humans are very good at determining the degree of roughness of different materials (Verrillo et al., 1999; Libouton et al., 2010), so the clear difference in the sensation of the materials validated the findings. Guest et al. (2011) found four significant sensory factors from using the TPT with different materials, where the primary sensory factor was the textural property of the material, and the other factors identified more complex tactile attributes such as aspects of wetness (cf. Moisture in the current results and Slip in Guest et al., 2011). Furthermore, significant differences were found between all the materials (apart from between Brush-Fur in Texture and Moisture). These findings demonstrate the sensitivity of the TPT to discriminate between the smaller differences in touch sensations. For the emotional factors, the differences occurred between the smoother (brush and fur) and rougher (sandpaper) materials: the brush and fur were rated as significantly less negative emotionally than the sandpaper, and there were no significant emotional differences between the brush and fur. Altogether, less is known about the emotional aspects of touch than the sensory aspects, previous work has shown variations on the pleasantness of touch where softer/smoother materials are rated as more pleasant (Major, 1895; Essick et al., 1999; Klöcker et al., 2012). The present study directly links the roughness sensory factor with the pleasantness emotional factor e.g., where sandpaper was rated as the roughest and most unpleasant. A major advantage of using the TPT is that it allows flexibility in the design of experiments so that the factors extracted represent a range of sensory and emotional tactile features of materials that can be compared against other variables such as skin site.

## Conclusions

We quantitatively investigated the description of touch to different skin sites, using different materials applied either by the participant or experimenter. The present study shows that the TPT is usable in a different language and culture, and extends the study of sensory as well as emotional aspects of touch. Touch to the arm and cheek skin evoked more affective assessment of the applied stimulus, differing from touch by the same material to the glabrous palm skin. We propose that these differences can be at least partly ascribed to the underlying neurophysiology of the skin afferents, specifically CT afferents, although further studies are needed to investigate this relationship directly. Skin containing CT afferents may preferentially signal affective aspects of touch, but it is clear that cognitive mechanisms will influence the perception of touch, as would the usage of the body site. We highlight differences in the functional properties of skin, with glabrous skin providing more exploratory and discriminative information, and hairy skin providing information on more emotional aspects of touch.

## Author contributions

All authors contributed to the conception of the work, design of the experiments, and in drafting/revising the manuscript. All authors have approved the final version and agree to be accountable for all aspects of the work. The data collection was carried out by Karin Saar and analyzed by Rochelle Ackerley, who also drafted manuscript. Rochelle Ackerley, Francis McGlone, and Helena Backlund Wasling interpreted the data.

### Conflict of interest statement

The authors declare that the research was conducted in the absence of any commercial or financial relationships that could be construed as a potential conflict of interest.
